# The Effect of Methyl-Derivatives of Flavanone on MCP-1, MIP-1β, RANTES, and Eotaxin Release by Activated RAW264.7 Macrophages

**DOI:** 10.3390/molecules29102239

**Published:** 2024-05-10

**Authors:** Małgorzata Kłósek, Anna Kurek-Górecka, Radosław Balwierz, Agnieszka Krawczyk-Łebek, Edyta Kostrzewa-Susłow, Joanna Bronikowska, Dagmara Jaworska, Zenon P. Czuba

**Affiliations:** 1 Department of Microbiology and Immunology, Faculty of Medical Sciences, Medical University of Silesia in Katowice, Jordana 19, 41-808 Zabrze, Poland; jbronikowska@sum.edu.pl (J.B.); djaworska@sum.edu.pl (D.J.); zczuba@sum.edu.pl (Z.P.C.); 2 Department of Community Pharmacy, Faculty of Pharmaceutical Sciences in Sosnowiec, Medical University of Silesia in Katowice, St Kasztanowa 3, 41-200 Sosnowiec, Poland; 3 Institute of Chemistry, University of Opole, Oleska 48, 45-052 Opole, Poland; radoslaw.balwierz@uni.opole.pl; 4 Department of Food Chemistry and Biocatalysis, University of Environmental and Life Sciences, Norwida 25, 50-375 Wrocław, Poland; agnieszka.krawczyk-lebek@upwr.edu.pl (A.K.-Ł.); edyta.kostrzewa-suslow@upwr.edu.pl (E.K.-S.)

**Keywords:** chemokines, flavanone, macrophages, inflammation, chemical structure

## Abstract

Chemokines, also known as chemotactic cytokines, stimulate the migration of immune cells. These molecules play a key role in the pathogenesis of inflammation leading to atherosclerosis, neurodegenerative disorders, rheumatoid arthritis, insulin-resistant diabetes, and cancer. Moreover, they take part in inflammatory bowel disease (IBD). The main objective of our research was to determine the activity of methyl-derivatives of flavanone, namely, 2′-methylflavanone (**5B**), 3′-methylflavanone (**6B**), 4′-methylflavanone (**7B**), and 6-methylflavanone (**8B**), on the releasing of selected cytokines by RAW264.7 macrophages activated by LPS. We determined the concentration of chemokines belonging to the CC chemokine family, namely, MCP-1, MIP-1β, RANTES, and eotaxin, using the Bio-Plex Magnetic Luminex Assay and the Bio-PlexTM 200 System. Among the tested compounds, only **5B** and **6B** had the strongest effect on inhibiting the examined chemokines’ release by macrophages. Therefore, **5B** and **6B** appear to be potentially useful in the prevention of diseases associated with the inflammatory process.

## 1. Introduction

Chemokines belong to a cytokine family known as chemotactic cytokines because they stimulate the migration of immune cells from the blood to peripheral tissues. In addition, they regulate cell proliferation, leukocyte activation and differentiation, and they affect the activation of adhesion molecules. Importantly, these molecules play a crucial role in the pathogenesis of inflammatory and autoimmune diseases and serve as key mediators of cancer cell proliferation [1,2,3]. They can be categorized based on their secretion into chemokines which are constitutively secreted by the bone marrow, thymus gland, secondary lymphoid organs, and non-lymphoid tissues such as the skin and mucosa. In contrast, pro-inflammatory chemokines are synthesized in response to stimulation by an infectious agent [4]. The names of cytokines originate from their specific function; however, chemokines are classified based on their structure, not their function. The nomenclature system for cytokines includes a four subfamily designation, namely, CXC, CX3C, XC, or CC, based on the number and position of cysteine residues at the beginning of their primary amino acid sequence [5]. The letter L with each chemokine signifies ‘ligand’, followed by a number representing the date of the first isolation of their gene [6]. IL-8 (CXCL8) belongs to the CXC subfamily, fractalkine (CX3CL1) to CX3C, and limfotactin alfa (XCL1) to XC. We analyzed selected cytokines belonging to the CC chemokine subfamily, the largest fourth subfamily group, which includes, among others, MCP-1 (CCL2), MIP-1β (CCL4), RANTES (CCL5), and eotaxin (CCL11). Monocytes, macrophages, and endothelial cells are the main source of chemokines. 

MCP-1 (Monocyte Chemoattractant Protein-1) primarily originates from monocyte/macrophages; however, it is also produced by fibroblasts, endothelial, or epithelial cells. MCP-1 regulates the migration and infiltration of monocytes, natural killer (NK) cells, and memory T lymphocytes. It has been implicated in the pathogenesis of several diseases characterized by monocyte infiltration, including atherosclerosis, rheumatoid arthritis, multiple sclerosis, insulin-resistant diabetes, or cancer [7,8]. MCP-1 triggers a significant migration of monocytes into the inflamed tissue and contributes to sustaining the inflammation. In patients with inflammatory bowel disease (IBD), besides the typical presence of intestinal macrophages, there is an additional reactive macrophage population along with elevated levels of monocyte chemoattractant protein 1 detected in the mucosa [9].

High amounts of MIP-1β (Macrophage Inflammatory Protein type 1 beta) are produced when monocytes are stimulated with LPS or IL-7 [10]. This cytokine is chemotactic for monocytes, T cells, and NK cells. RANTES (Regulated on Activation Normal T cell Expressed and Secreted) is produced by macrophages, fibroblasts, endothelial cells, T cells, or platelets [11]. The expression of RANTES has been associated with inflammatory disorders, including asthma, atopic dermatitis, atherosclerosis, arthritis, or allogeneic transplant rejection [12,13]. Eotaxins, members of the chemokine family, comprise eotaxin-1 (CCL11), eotaxin-2 (CCL24), and eotaxin-3 (CCL26). These proteins have the ability to attract and activate cells that bear the CCR3 receptor. Chemokines including eotaxins, CCL2, CCL5, CCL17, and CCL22 are involved in allergic asthma [14]. The expression of eotaxins is induced by pro-inflammatory cytokines and enables the migration of macrophages, dendritic cells, basophils, or eosinophils. A study revealed that the levels of CCL11 in patients with rheumatoid arthritis were notably higher before the disease developed compared to healthy group. After the onset of rheumatoid arthritis, these levels increased even more [15]. 

The sensitivity of immune cells to a given cytokine is determined by the chemokine G-protein-coupled receptor specific for it, which is present on their surface. Significant amounts of cytokines, including chemokines, are released during inflammation. The inflammatory process can be triggered by bacterial infection, with lipopolysaccharide (LPS) from Gram-negative bacteria serving as a potent inducer of cytokine production [16]. The inflammatory response triggered by LPS may lead to potentially fatal septic shock. The RAW264.7 macrophage line is a suitable model for in vitro cytokine research. 

We aim to explore chemical compounds of natural origin or obtained by chemical synthesis that could modulate the body’s immune response to the inflammatory process. Our primary objective is to assess the activity of methyl-derivatives of flavanone by measuring selected chemokines released by LPS-activated RAW264.7 macrophages. Specifically, we investigated the effects of 2′-methylflavanone (**5B**), 3′-methylflavanone (**6B**), 4′-methylflavanone (**7B**), and 6-methylflavanone (**8B**) on the release of MCP-1, MIP-1β, RANTES, and eotaxin by macrophages activated by LPS. In a previous study, we demonstrated that methyl-derivatives of flavanone possess in vitro anti-inflammatory activity and can modulate the release of IL-1β, IL-6, IL-12p40, IL-12p70, and TNF-α by activated macrophages [17]. 

**Figure 1:**
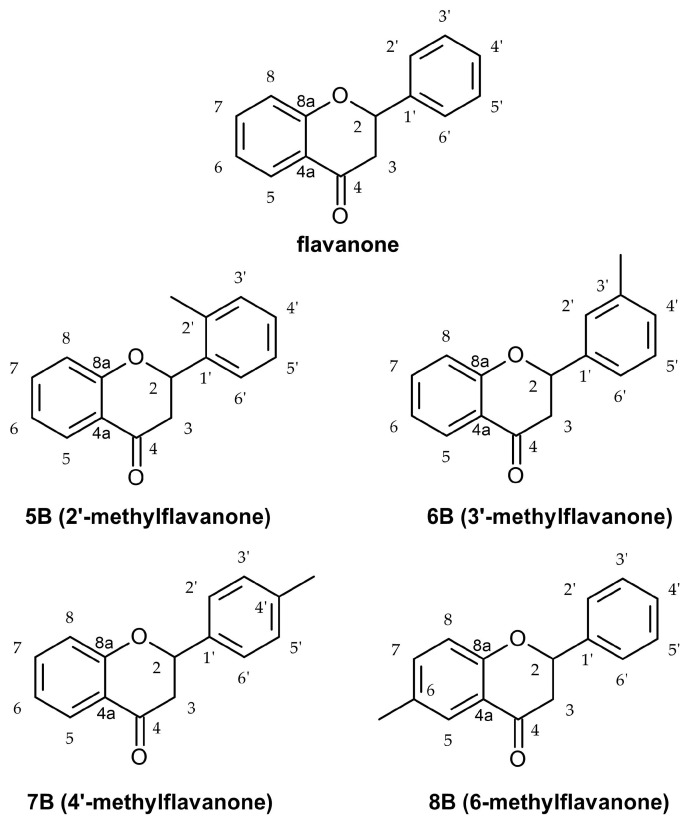
Structure of flavanone and methyl-derivatives of flavanone **5B**–**8B**.

In our study, we show for the first time that methyl-derivatives of flavanone can modulate chemokines released by LPS-stimulated macrophages. We demonstrate that 2′-methylflavanone (**5B**) and 3′-methylflavanone (**6B**) exhibit the strongest inhibitory activity among the tested flavanone derivatives in reducing the concentration of MCP-1, MIP-1β, RANTES, and eotaxin compared to the control and the core flavanone structure. Structure of flavanone and methyl-derivatives of flavanone used in the experiment is shown in Figure 1.

## 2. Results

### 2.1. The Effect of the Flavanone, 2′-Methylflavanone (***5B***), 3′-Methylflavanone (***6B***), 4′-Methylflavanone (***7B***), and 6-Methylflavanone (***8B***) on the Release of MCP-1 by Macrophages

**Figure 2:**
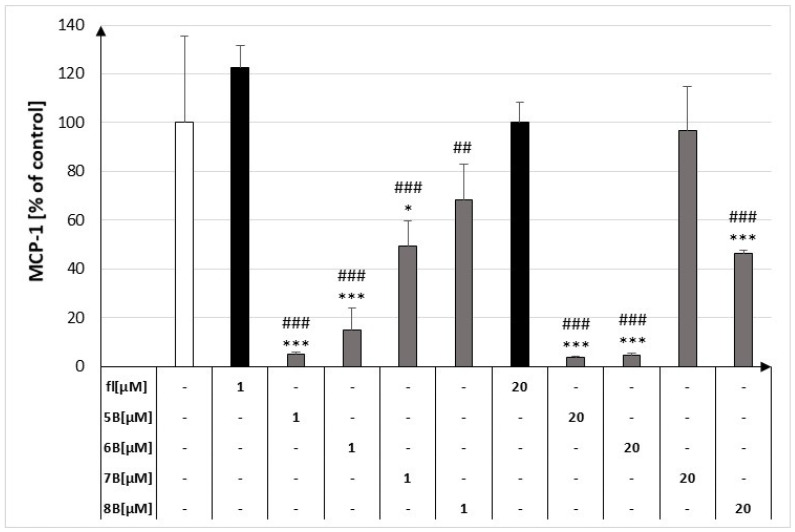
Concentrations of MCP-1 in culture supernatants of RAW264.7 cell lines in the presence of flavanone (fl), 2′-methylflavanone (**5B**), 3′-methylflavanone (**6B**), 4′-methylflavanone (**7B**), and 6-methylflavanone (**8B**); ## *p* < 0.01; ### *p* < 0.001 compared to flavanone; * *p* < 0.05, *** *p* < 0.001 compared to control (control cells and compound incubation with LPS).

All tested compounds significantly decreased the concentration of MCP-1 released by RAW 264.7 cells. The greatest inhibitory effect was observed for compounds **5B** and **6B**. Compound **5B** at 1 μM reduced the concentration of MCP-1 to 4.89% ± 0.96% compared to the control and at 20 μM to 3.77% ± 0.58% compared to the control. However, compound **6B** reduced the concentration of MCP-1 to 14.89% ± 9.20% and 4.73% ± 0.86% compared to the control, respectively (Figure 2).

### 2.2. The Effect of the Flavanone, 2′-Methylflavanone (***5B***), 3′-Methylflavanone (***6B***), 4′-Methylflavanone (***7B***), and 6-Methylflavanone (***8B***) on the Release of MIP-1β by Macrophages

**Figure 3:**
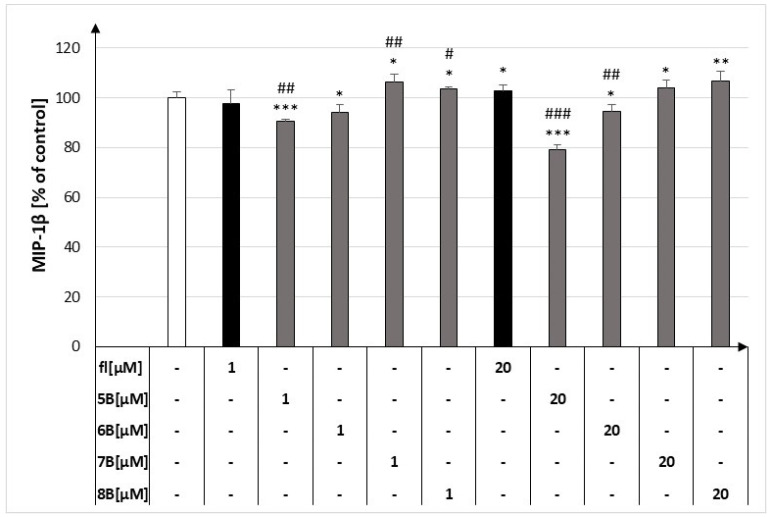
Concentrations of MIP-1β in culture supernatants of RAW264.7 cell lines in the presence of flavanone (fl), 2′-methylflavanone (**5B**), 3′-methylflavanone (**6B**), 4′-methylflavanone (**7B**), and 6-methylflavanone (**8B**); # *p* < 0.05, ## *p* < 0.01, ### *p* < 0.001 compared to flavanone; * *p* < 0.05, ** *p* < 0.01, *** *p* < 0.001 compared to control (control cells and compound incubation with LPS).

At both concentrations, compound **5B** decreased the release of MIP-1β by macrophages to 90.46% ± 0.80% and 79.32% ± 1.82% compared to the control, respectively. Compound **6B** at both concentrations showed a weaker inhibitory effect than **5B**. The other compounds showed no inhibitory effect on this labelled cytokine compared to the control (Figure 3).

### 2.3. The Effect of the Flavanone, 2′-Methylflavanone (***5B***), 3′-Methylflavanone (***6B***), 4′-Methylflavanone (***7B***), and 6-Methylflavanone (***8B***) on the Release of RANTES by Macrophages

**Figure 4:**
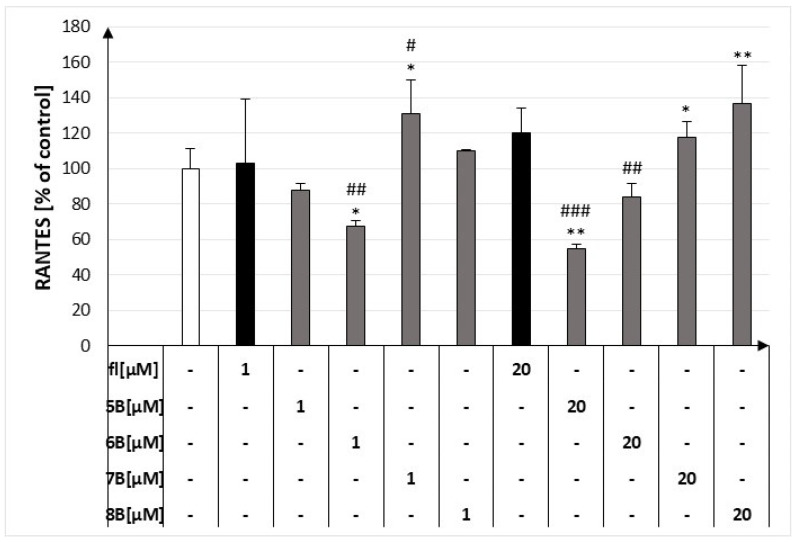
Concentrations of RANTES in culture supernatants of RAW264.7 cell lines in the presence of flavanone (fl), 2′-methylflavanone (**5B**), 3′-methylflavanone (**6B**), 4′-methylflavanone (**7B**), and 6-methylflavanone (**8B**); # *p* < 0.05, ## *p* < 0.01, ### *p* < 0.001 compared to flavanone; * *p* < 0.05, ** *p* < 0.01 compared to control (control cells and compound incubation with LPS).

At the higher concentration, compound **5B** most strongly inhibited the release of RANTES by macrophages from 92.25% ± 10.39% to 55.05% ± 2.42%. However, compound **7B** at both concentrations and compound **8B** at 20 μM increased the release of RANTES by macrophages to 131.16% ± 19.21%, 117.49% ± 9.02%, and 137.02% ± 21.19%, respectively (Figure 4).

### 2.4. The Effect of the Flavanone, 2′-Methylflavanone (***5B***), 3′-Methylflavanone (***6B***), 4′-Methylflavanone (***7B***), and 6-Methylflavanone (***8B***) on the Release of Eotaxin by Macrophages

**Figure 5:**
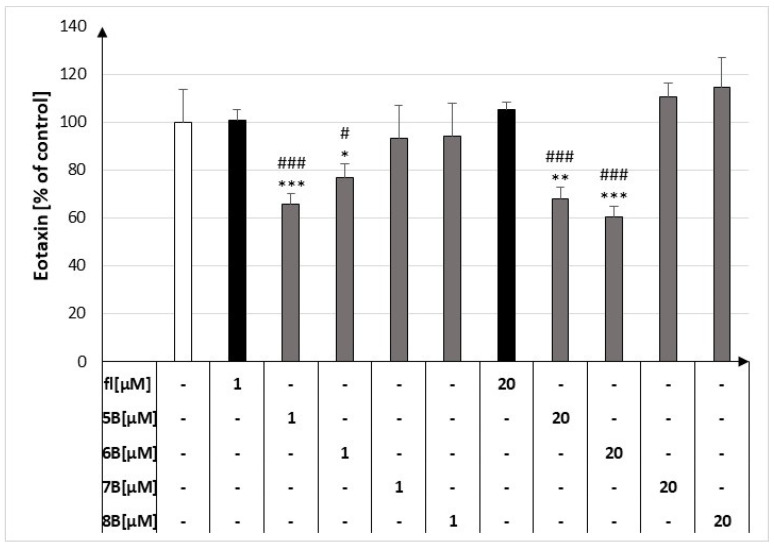
Concentrations of eotaxin in culture supernatants of RAW264.7 cell lines in the presence of flavanone (fl), 2′-methylflavanone (**5B**), 3′-methylflavanone (**6B**), 4′-methylflavanone (**7B**), and 6-methylflavanone (**8B**); # *p* < 0.05, ### *p* < 0.001 compared to flavanone; * *p* < 0.05, ** *p* < 0.01, *** *p* < 0.001 compared to control (control cells and compound incubation with LPS).

At 1 μM and 20 μM, compound **5B** decreased eotaxin released by macrophages to 65.71% ± 4.58% and 68.09% ± 4.69% compared to the control, respectively. The mentioned results are depicted in Figure 5. Compound **6B** at 1 μM and 20 μM decreased eotaxin released by macrophages to 76.85% ± 5.70% and 60.37% ± 4.26% compared to the control, respectively.

The results of pairwise comparisons for all tested compounds on the production of the tested cytokines in LPS-stimulated RAW264.1 cells are presented in Table 1 and in the Supplementary Materials (Tables S1–S4).

### 2.5. HCA of All Obtained Data Based on the Average Content of the Effect of Methyl-Derivatives of Flavanone Regarding the Behavior of Cytokines

**Figure 6:**
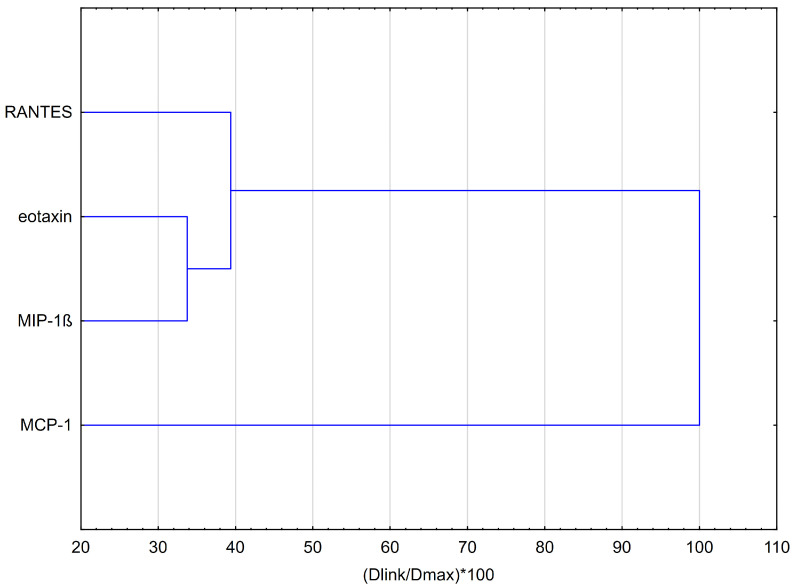
Dendrogram obtained via the HCA of data regarding the behavior of RANTES, exotoxin, MIP-1β, and MCP-1 secretion by HGF-1 fibroblasts stimulated by LPS following treatment with 2′-methylflavanone (**5B**), 3′-methylflavanone (**6B**), 4′-methylflavanone (**7B**), and 6-methylflavanone (**8B**) at different concentrations. Dlink is the distance between two clusters (linkage distance). Dmax is the maximum possible distance between clusters.

**Figure 7:**
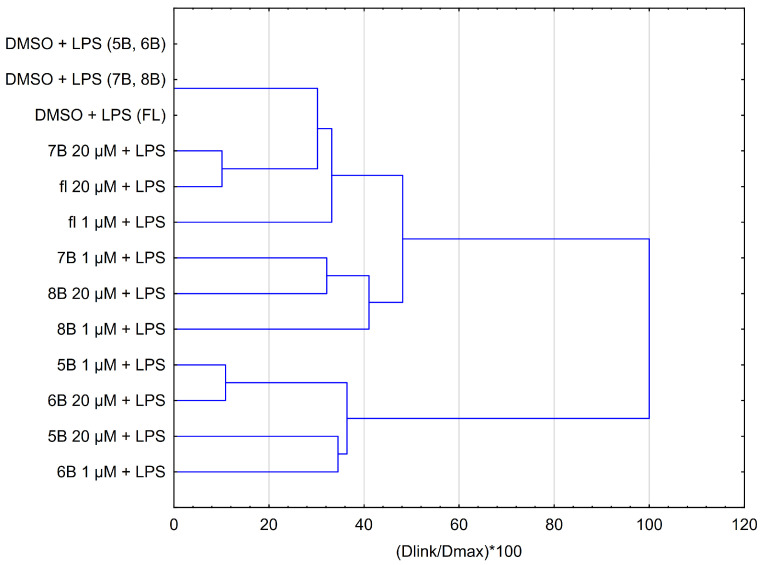
Dendrogram obtained by the HCA of all obtained data based on the influence on chemokines by the average content of the 2′-methylflavanone (**5B**), 3′-methylflavanone (**6B**), 4′-methylflavanone (**7B**), and 6-methylflavanone (**8B**) at different concentrations; K KDMO—control line consists of DMSO (dimethyl sulfoxide) at a final concentration of 0.1%. Dlink is the distance between two clusters (linkage distance). Dmax is the maximum possible distance between clusters.

Hierarchical clustering analysis (HCA, using Euclidean distances) and principal component analysis (PCA) were employed to analyze the impact of methyl-derivatives of flavanone, namely, 2′-methylflavanone (**5B**), 3′-methylflavanone (**6B**), 4′-methylflavanone (**7B**), and 6-methylflavanone (**8B**), on the concentrations of chemokines evaluated in RAW264.7 macrophages stimulated by LPS. The results obtained from the HCA are presented in Figure 6 and Figure 7. The HCA relied on Euclidean distance calculations between sets. The resulting chemokine dendrogram (Figure 6) revealed that the entire dataset could be grouped into three main clusters. The flavanones have a similar effect on eotaxin and MIP-1β. Methyl flavanones are modestly inhibitory of RANTES release. Notably, the MCP-1 cytokine does not form clusters with the other cytokines, indicating a lack of similarity in properties with other cytokines or a different mode of action of methyl-derivatives of flavanone on this cytokine. 

### 2.6. HCA of All Obtained Data Based on the Influence on Chemokines by the Average Content of the Methyl-Derivatives of Flavanone

The results obtained from the HCA are presented in Figure 7. There are similarities between compounds **5B** and **6B** at both concentrations, as illustrated by their comparable effects (Figure 7). Specifically, the impact of compound **5B** at 1 μM closely resembles that of compound **6B** at 20 μM. Likewise, compounds **7B** (1 μM) and **8B** (20 μM) exhibit similar characteristics. Moreover, **7B** at 20 μM has a similar activity to flavanone at 20 μM.

### 2.7. PCA Score Plot of All Obtained Data Based on the Average Content of the Effect of the Methyl-Derivatives of Flavanone

**Figure 8:**
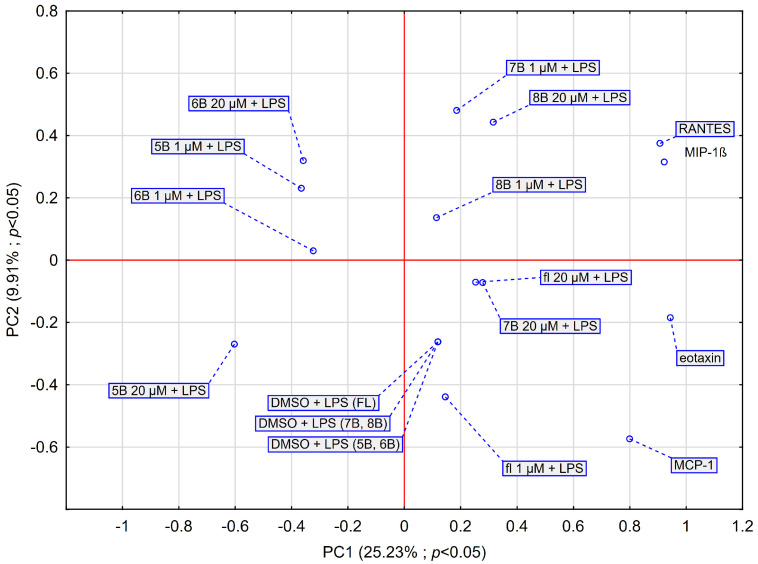
PCA score plot of all obtained data based on the average content of the effect of the 2′-methylflavanone (**5B**), 3′-methylflavanone (**6B**), 4′-methylflavanone (**7B**), and 6-methylflavanone (**8B**) at different concentrations on chemokines. PC—principal component; DMSO—control line consists of DMSO (dimethyl sulfoxide) at a final concentration 0.1%. Abbreviations: fl—flavanone.

The results from the PCA are shown in Figure 8. The PCA score plot was generated considering the average impact of two concentrations of methyl-derivatives of flavanone on chemokines (Figure 8). The first two principal components (PCs) explained 33.15% of the variance in the data (23.3% for PC1 and 9.9% for PC2). The results obtained for MIP-1β and RANTES are very similar to each other, eotaxin shows less similarity, while MCP-1 completely diverges from the other chemokines, which confirms the results obtained earlier. The PCA results also indicate that the effects of flavanone at 20 μM, compound **7B** at 20 μM, and compound **8B** at 1 μM are similar. Compound **7B** at 1 μM and **8B** in both concentrations interact more with MIP-1β and RANTES than the other tested compounds. The data obtained are consistent with those shown in Figure 1, Figure 2, Figure 3 and Figure 4). In contrast, flavanone at 20 μM and **7B** at 20 μM interact more with eotaxin. In terms of their effects on the chemokines we labelled, compound **6B** at both concentrations and **5B** at 1 μM appear to be similar. Compounds **5B** and **6B** show a different effect to compounds **7B** and **8B**. Furthermore, compound **5B** at 20 μM acts quite differently from the other compounds we tested.

## 3. Discussion

Biotransformation is a new strategy to create bioactive flavonoids for medicinal purposes. The correlation between chemical structure and biological activity allows the creation of biotechnological strategies to produce novel bioactive compounds with enhanced biological potential [18,19]. Sisa et al. confirmed that methoxylated flavonoids take part in stress protection and play a role in chemical defense in plants. It was highlighted that O-methylated flavonoids exhibit stronger anticancer action than their hydroxylated forms [20].

In our study, we used lower concentrations of methyl-derivatives of flavanone to align with values observed in vivo. The bioavailability of polyphenols is directly linked to their properties. The assessment of polyphenol bioavailability often revolves around their maximum plasma concentration (Cmax). Factors such as absorption in the gastrointestinal tract, plasma transport, and metabolism influence the bioavailability of phenolic compounds. Polyphenols typically undergo digestion by intestinal enzymes or colonic bacteria before absorption occurs [21]. The study of Gardana C. has shown that maximum plasma concentrations of hesperetin and naringenin after the ingestion of 300 mL of blood orange juice were in the range of 15–200 and 15–80 ng/mL, respectively [22]. 

Among the newly synthesized methyl flavanone derivatives, compounds **5B** and **6B** exhibited no cytotoxicity within the concentration range of 1 μM–20 μM, unlike **7B** and **8B**, as demonstrated in our previous research [17]. In the research hypothesis, we assumed that the newly synthesized derivatives would be already active at low concentrations. For comparison, we used a concentration of 20 µM as one of the lower concentration ranges in which flavonoids affect chemokines [23].

Flavanones belong to flavonoids which are derivatives of 2-phenylbenzo-γ-pyrone. The common part in the chemical structure of all flavonoids is a carbon skeleton based on the flavone system (C6-C3-C6), formed of two benzene rings (A and B) linked with a heterocyclic pyran or pyrone ring C. The individual flavonoids differ from each other by the substituents in the rings, which are formed as a result of hydroxylation, methylation, acylation, and glycosidation with mono- or oligosaccharides at various positions of the rings [24].

The relationship between the chemical structure of flavonoids and their antioxidative activity influencing their medicinal properties is known [25]. 

The antioxidative activity of flavonoids is linked to their chemical structure. The *ortho*-dihydroxy group in the B ring is connected with the ability to produce the free radical scavenging effect and is responsible for the stability of phenoxyl radical. The double bond between C2 and C-3 carbon as well as the 4-oxo-group in the C ring cause the dislocation of an electron in the B ring. Moreover, the presence of hydroxyl group near C-3 and C-5 carbon as well as the 4-oxo group in the A and C ring enhance the free radical scavenging effect. Additionally, the presence of the methoxyl group may have an impact on the antioxidative properties. The methoxyl groups in the C-3 position by a steric hindrance may decrease the antioxidant activity. Naringin and naringenin, belonging to flavanones, demonstrate low antiradical activity. Therefore, the impact of methoxyl groups on antiradical properties seems interesting. The slight antioxidant activity for hesperetin indicates the interaction of methoxyl substituted in a certain position on antioxidant activity [26,27].

The mechanism of the antioxidative activity of flavonoids involves the inhibition of the enzyme activity, e.g., xanthine oxidase, oxidase of reduced nicotinamide adenine dinucleotide phosphate (NADPH), and, as a consequence, the inhibition of generating reactive oxygen species. In addition, flavonoids cause chelating ions of metals which take part in the creation of free radicals, that interrupt lipid peroxidation by scavenging reactive oxygen species as well as synergistic effects with other antioxidants. The anti-inflammatory activity of flavonoids may result in their antioxidant activity. The mechanism of anti-inflammatory action of flavonoids, for example, naringenin, involves the inhibition of the activity of 5-lipoxygenase (5-LOX) and cyclooxygenase (COX, especially COX-2) [28].

Moreover, flavonoids exhibit an anti-cancerogenic mechanism which includes the inhibition of tyrosine kinase C. The mentioned enzyme takes part in the growth and proliferation of cancer cells [29]. Among flavanones, hesperitin and naringenin exhibit antiproliferative action with regard to the breast cancer estrogen receptor [30]. 

In the conducted study, we decided to evaluate the impact of new methyl-derivatives of flavanones on chemokines. Chemokines not only enhance the migration of immune cells into inflamed tissues but also participate in the pathogenesis of diseases characterized by chronic inflammation, including neurodegenerative disorders, cardiovascular diseases, autoimmune disorders, diabetic complications, and cancer [31,32]. Tissue damage or bacterial infection results in the recruitment of immune cells to the site of the ongoing inflammatory response. Neutrophils, mast cells, resident macrophages, and recruited monocytes further differentiate into macrophages, secreting inflammatory mediators, including pro-inflammatory cytokines and chemokines. These mediators enter the bloodstream and attract other immune cells. Cardiovascular mediators generated from inflammatory cells and damaged tissues regulate the inflammatory response. These mediators include vasoactive amines such as histamine and serotonin, peptides (e.g., bradykinin), and eicosanoids (e.g., prostaglandins, leukotrienes, and thromboxanes) [33,34]. MCP-1 is involved in the initial steps of the pathogenesis of atherosclerotic cardiovascular disease and neurodegenerative disorders. It is produced in response to LPS, oxidized low-density lipoproteins (oxLDLs), and proinflammatory cytokines such as IL-1β, TNF-α, or IFN-γ [35]. CCL2 also shows a good correlation with the strength of the inflammatory response. However, these potential biomarkers are less useful for individual patients due to their lack of specificity [36]. 

Machura et al. have shown that the concentration of RANTES was significantly higher in children with atopic asthma as compared to children in the control group [37]. Grimm M.C. et al. have shown that the tissue of ulcerative colitis exhibits strong mRNA expression for chemokines, as observed with MIP-1α and RANTES [38]. Numerous studies have shown that flavonoids, including flavanones, possess a broad range of biological activities including anti-inflammatory, antioxidant, anticancer, neuroprotective, and anti-atherosclerotic properties [39,40,41,42,43]. Natural or synthetic compounds show an inhibitory effect on MCP-1 production. Kowalski at al. examined the effect of kaempferol (3,4′,5,7-tetrahydroxyflavone) on the production of MCP-1 in the J774.2 macrophage cell line stimulated with LPS [44]. Kaempferol at both concentrations used (10 μM and 30 μM) reduced the secretion of MCP-1 released by macrophages, and at a 30 μM, it significantly decreased the number of copies of MCP-1 mRNA. Hada et al. showed that fisetin (3,3′,4′,7-tetrahydroxyflavone) suppressed mRNA expression levels of MCP-1, IL-1β, and iNOS and suppressed NO production in LPS-treated macrophages [45]. Liu et al. evaluated the effect of naringin (5,7,4′-trihydroxyflavanone-7-O-neohesperidoside), a glycoside of naringenin, on chemokine expression in LPS-induced RAW 264.7 macrophages [46]. They demonstrated that naringin reduces IL-8, MCP-1, and MIP-1α secretion in LPS-induced RAW 264.7 macrophages. Moreover, this flavanone reduces the mRNA expression of the tested chemokines, possibly by blocking the activation of the NF-κB and MAPK signaling pathways. Hsueh et al. showed that naringin exhibits an anti-atherosclerotic effect by downregulating the TNF-α-induced expression of cell adhesion molecules and chemokines like fractalkine/CX3CL1, MCP-1, and RANTES in human umbilical vein endothelial cells (HUVECs) [47]. 

The hierarchical cluster analysis (HCA) and principal component analysis (PCA) of our results showed that the MCP-1 cytokine is completely different from the others we analyzed. Also, the compounds we tested have a different effect on this cytokine. Among the examined compounds, 2′-methylflavanone (**5B**) and 3′-methylflavanone (**6B**) achieved a similarly strong inhibition of MCP-1 production by activated macrophages compared to the control and flavanone. Interestingly, compound **7B** at a higher concentration causes an increase in the concentration of MCP-1 compared to the control. Perhaps the methyl group in the 2′ and 3′ positions influences the higher activity of the compound compared to the methyl group in the 4′ and 6 positions. Even in low concentrations, flavonoids exhibit a biological and immunomodulatory effect. Ha S.H. demonstrated that apigenin at concentrations of 5 μM and 10 μM significantly inhibited NO production in BV-2 murine microglia cells [48]. Kao T-K. showed that luteolin significantly inhibited NO production in rat primary microglia and BV-2 cells [49]. Furthermore, luteolin was found to significantly inhibit the proinflammatory cytokines IL-1β and TNF-α released by lipopolysaccharide (LPS)/interferon γ (IFN-γ)-induced cells at concentrations of 15 μM and 20 μM, respectively. In our study compound **7B** has no effect on MCP-1, whereas **8B** has an effect at 20 μM concentrations used in comparison to control.

In addition, flavanone without substituents at both concentrations demonstrates slight differences in its ability to release MCP-1 by macrophages. Moreover, compound **7B** reacts most strongly with free radicals, as shown in our previous publication [17]. Therefore, free radical systems in the cell may be affected. Applying a slightly higher concentration of compound **7B** abolishes the inhibitory effect on MCP-1. Bruser L. et al. have shown that cannflavin A, a prenylated flavonoid in opposition to quercetin, kaempferol, and luteolin, did not inhibit IL-1β-induced MCP-1 mRNA and protein expression in human coronary artery endothelial cells (HCAECs). MCP-1 secretion was higher for 5 μM than for 20 μM after cannflavin A treatment [50]. 

The PCA and HCA revealed that the MIP-1β, eotaxin, and RANTES are very similar to each other, unlike MCP-1. Flavanone (20 μM), 4′-methylflavanone (**7B**) at 20 μM, and 6-methylflavanone (**8B**) at 1 μM had no significant inhibitory effect on eotaxin. However, a statistically significant inhibitory effect on eotaxin was observed for 2′-methylflavanone (**5B**) and 3′-methylflavanone (**6B**). Jeon et al. demonstrated that eupatilin (5,7-dihydroxy-3′,4′,6′-trimethoxyflavone), a bioactive component of *Artemisia asiatica*, significantly inhibited the expression of eotaxin in bronchial epithelial cells stimulated with TNF-α [51]. Eupatilin also inhibited MAPK (mitogen-activated protein kinase) activity. Jayaprakasam showed that liquiritigenin, isoliquiritigenin, and 7,4′-dihyroxyflavone, contained in the herbs *Glycyrrhizae uralensis,* inhibited eotaxin-1 in a dose-dependent manner [52]. 

The PCA showed that **8B** (1 μM), **7B** (1 μM), and **8B** (20 μM) have a more significant impact on RANTES and MIP-1β than the other examined compounds in stimulating the release of these chemokines. In contrast, compounds **5B** and **6B** significantly reduce the concentration of these cytokines. However, **5B** at 20 μM behaves differently from the rest of the compounds we studied; it does not form a characteristic cluster. It is likely the most active compound among those studied. Baicalin, a flavone glycoside found in *Scutellaria baicalensis* at 10–50 μM, significantly decreases the production of RANTES, MIP-1α, MIP-1β, MIP-2, IL-6, granulocyte colony-stimulating factor (G-CSF), and vascular endothelial growth factor (VEGF) in LPS-stimulated RAW264.7 cells [53]. Some flavanones and their derivatives possess anticancer activities. Tacotanina, a 3′,4′-dihydroxy-5,7-dimethoxyflavonone isolated from the leaves of *Chromolaena tacotana*, inhibits the cell proliferation of triple-negative human breast cancer cell lines MDA-MB-231 and MCF-7 [54]. The scientists noted a significant inactivation of the anti-apoptotic protein Bcl-2 and the activation of effector caspase-3 and/or 7 within the breast cancer cells. Another flavanone, 2′-hydroxyflavanone, inhibits the progression of the human pancreatic cancer cell lines BxPC-3 and MIA PaCa-2 [55]. The authors showed that 2HF induces apoptosis and arrests cell cycle in these cells. 

Summarizing, we confirm that the chemical structure of compounds determines their biological properties. Among the tested methyl-derivatives of flavanone, compounds **5B** and **6B** had the strongest effect on inhibiting the release of the selected chemokines MCP-1, MIP-1β, RANTES, and eotaxin by activated RAW264.7 macrophages. Therefore, **5B** and **6B** appear to be potentially useful in the prevention of diseases associated with the inflammatory process.

## 4. Materials and Methods

### 4.1. General Procedure for the Synthesis of Methyl-Flavanones

The synthesized methyl-derivatives of flavanone were characterized using spectroscopic methods along with NMR and MS and included in Supplementary Materials in previous articles [17,56,57,58,59]. 

### 4.2. Cell Culture

In our study, we used RAW 264.7, a mouse peritoneal macrophage cell line purchased from ATCC (American Type Culture Collection, Manassas, VA, USA). These cells were derived from a tumor in a male mouse that was caused by the Abelson murine leukemia virus. Cell cultures were maintained at a constant temperature of 37 °C in an environment with 5% CO_2_ and full humidity. Macrophages were cultured in a medium containing DMEM supplemented with 10% heat-treated fetal bovine serum (FBS), along with 100 IU/mL penicillin and 100 μg/mL streptomycin. Any macrophage cells adhered to the surface were detached by scraping and then suspended for further experimentation. The concentration of the cell suspension was determined using a Bürker chamber under microscopy. RAW264.7 cells were seeded at a density of 1 × 10^6^ cells/mL in 96-well plates, with or without LPS (200 ng/mL), along with methyl-derivatives of flavanone, and incubated for 24 h.

### 4.3. Quantification of MCP-1, MIP-1β, RANTES, and Eotaxin Concentrations

Chemokines released from the RAW264.7 cell line after stimulation with methyl-derivatives of flavanone (1–20 μg/mL) were analyzed in the culture supernatant. A multiplex assay, specifically the Bio-Plex Human Cytokine Panel from BIO-RAD, was used for cytokine detection. This assay utilized the Bio-Plex (Version 5.0) 200 System based on xMAP technology (BIO-RAD Laboratories Inc., Hercules, CA, USA). The native cells (1 × 10^6^/mL) were stimulated by 2′-methylflavanone (**5B**), 3′-methylflavanone (**6B**), 4′-methylflavanone (**7B**), and 6-methylflavanone (**8B**) with LPS incubation for 24 h. The supernatant was mixed with magnetic beads linked to antibodies, left to sit for half an hour, and then washed. After that, special antibodies labeled with biotin and streptavidin-phycoerythrin compounds were introduced and allowed to incubate for another 30 min. Any leftover streptavidin was washed away. Lastly, the Bio-Plex 200 System was employed to identify the specific cytokines attached to the beads. Fluorescence levels were recorded using BIO-RAD’s Bio-Plex Manager software, Version 5.0 [60,61].

### 4.4. Statistical Analysis

The results of cytokine determination represent means ± SD obtained from three independent experiments (n = 3). The statistical analyses were performed using STATISTICA 13.1 software (StatSoft Inc., Tulsa, OK, USA). A one-way ANOVA was used to compare the effect of methyl-derivatives of flavanones among the tested compounds and control, as well as among the tested compounds and flavanones. Statistical significance was calculated using Fisher’s LSD test, with significance considered when *p* < 0.05. Moreover, the results were analyzed using HCA and PCA. The HCA was performed using full linkage using Euclidean distance. The PCA model was estimated using the NIPALS iterative algorithm. 

## 5. Conclusions

Among the compounds analyzed, **5B** and **6B** showed the strongest effect in inhibiting the release of the examined chemokines by macrophages. Therefore, **5B** and **6B** appear to be potentially useful in the prevention of diseases associated with the inflammatory process including, but not limited to, the lining of the intestinal mucosa. Our study showed that the compound differing the most in terms of activity is 2′-methylflavanone (**5B**) at 20 μM. It may be the most active compound among all those examined. This compound significantly inhibits MCP-1, MIP-1β, RANTES, and eotaxin released by RAW264.7 macrophages activated by LPS. Further research is needed to demonstrate the possible anticancer activity of the tested compound.

## Figures and Tables

**Table 1:** The effect of methyl-derivatives of flavanone on the production of MCP-1, MIP-1β, RANTES, and eotaxin in comparison to control in LPS-stimulated RAW264.1 cells (n = 3). Statistical significance was analyzed using Fisher’s LSD test. Results marked in red are statistically significant in Fisher’s LSD test. Multivariate Tests of Significance (F = 8.20, *p* < 0.05).

Sample	MCP-1			MIP-1β			RANTES			eotaxin		
	AVG [%]	SD	*p*	AVG [%]	SD	*p*	AVG [%]	SD	*p*	AVG [%]	SD	*p*
Control	100	35.69		100	2.33		100	11.69		100	13.8	
**5B** 1 μM	4.890506	0.96	0.000009	90.46556	0.8	0.000497	87.65191	4.21	0.329811	65.70678	4.58	0.000505
**5B** 20 μM	3.775331	0.58	0.000008	79.32471	1.82	0.000000	55.04602	2.42	0.001264	68.08773	4.69	0.001030
**6B** 1 μM	14.8888	9.2	0.000043	94.25956	2.91	0.024173	67.5061	2.92	0.014710	76.85006	5.7	0.012593
**6B** 20 μM	4.730866	0.86	0.000009	94.51437	2.7	0.30536	84.14098	7.56	0.213420	60.37109	4.26	0.000100
**7B** 1 μM	49.2387	10.6	0.007050	106.2924	3.05	0.014348	131.1568	19.21	0.018808	93.02393	13.85	0.426578
**7B** 20 μM	96.91125	18.03	0.860102	104.1507	2.93	0.095306	117.4937	9.02	0.171286	110.4894	5.85	0.235467
**8B** 1 μM	68.51843	14.47	0.081197	103.4281	1.04	0.164727	110.2431	0.53	0.417540	94.2095	13.48	0.508481
**8B** 20 μM	46.43181	1.31	0.004759	106.858	3.7	0.008244	137.0226	21.19	0.006214	114.4726	12.62	0.105781
Flavanone 1 μM	122.7477	8.91	0.201345	97.53397	5.67	0.313226	103.3669	35.97	0.788692	100.7151	4.66	0.934649
Flavanone 20 μM	100.3554	8.19	0.983816	102.8635	2.4	0.243199	120.4037	13.61	0.112843	105.2264	3.19	0.550337

## Data Availability

Data are contained within the article.

## Supplementary Materials

The following supporting information can be downloaded at: https://www.mdpi.com/article/10.3390/molecules29102239/s1, Table S1: Results of pair-wise comparisons of test compounds on the production MCP-1 in compared to control and flavanone in LPS stimulated RAW264.1 cells (n = 3). Statistical significance was analysed using Fisher’s LSD test. Results marked in red are statistically significant in Fisher’s LSD test. Multivariate Tests of Significance (F = 8.20, *p* < 0.05); Table S2: Results of pair-wise comparisons of test compounds on the production MIP-1β in compared to control and flavanone in LPS stimulated RAW264.1 cells (n = 3). Statistical significance was analysed using Fisher’s LSD test. Results marked in red are statistically significant in Fisher’s LSD test. Multivariate Tests of Significance (F = 8.20, *p* < 0.05); Table S3. Results of pair-wise comparisons of test compounds on the production RANTES in compared to control and flavanone in LPS stimulated RAW264.1 cells (n = 3). Statistical significance was analysed using Fisher’s LSD test. Results marked in red are statistically significant in Fisher’s LSD test. Multivariate Tests of Significance (F = 8.20, *p* < 0.05); Table S4: Results of pair-wise comparisons of test compounds on the production eotaxin in compared to control and flavanone in LPS stimulated RAW264.1 cells (n = 3). Statistical significance was analysed using Fisher’s LSD test. Results marked in red are statistically significant in Fisher’s LSD test. Multivariate Tests of Significance (F = 8.20, *p* < 0.05).
