# Supplementary material for: The Effect of Methyl-Derivatives of Flavanone on MCP-1, MIP-1β, RANTES, and Eotaxin Release by Activated RAW264.7 Macrophages
Source: Molecules. 2024 May 10;29(10):2239. doi: 10.3390/molecules29102239 (PMC11123782; doi:10.3390/molecules29102239)
Supplement: Supplementary file 1 [file molecules-29-02239-s001.zip › molecules-2935294-supplementary.pdf]

molecules

MDPI

Supplementary Materials

The Effect of Methyl-Derivatives of Flavanone on MCP-1, MIP-1β, RANTES, and Eotaxin Release by Activated RAW264.7 Macrophages

Małgorzata Kłósek [1,*], Anna Kurek-Górecka [2,*], Radosław Balwierz [3], Agnieszka Krawczyk-Łebek [4], Edyta Kostrzewa-Susłow [4], Joanna Bronikowska [1], Dagmara Jaworska [1] and Zenon P. Czuba [1]

1 Department of Microbiology and Immunology, Faculty of Medical Sciences, Medical University of Silesia in Katowice, Jordana 19, 41-808 Zabrze, Poland; jbronikowska@sum.edu.pl (J.B.); djaworska@sum.edu.pl (D.J.); zczuba@sum.edu.pl (Z.P.C.)

2 Department of Community Pharmacy, Faculty of Pharmaceutical Sciences in Sosnowiec, Medical University of Silesia in Katowice, St Kasztanowa 3, 41-200 Sosnowiec, Poland

3 Institute of Chemistry, University of Opole, Oleska 48, 45-052 Opole, Poland; radoslaw.balwierz@uni.opole.pl

4 Department of Food Chemistry and Biocatalysis, University of Environmental and Life Sciences, Norwida 25, 50-375 Wrocław, Poland; agnieszka.krawczyk-lebek@upwr.edu.pl (A.K.-Ł.); edyta.kostrzewa-suslow@upwr.edu.pl (E.K.-S.)

* Correspondence: mklosek@sum.edu.pl (M.K.); akurekgorecka@sum.edu.pl (A.K.-G.); Tel./Fax: +48-322-722-554 (M.K.)

Citation: Kłósek, M.; Kurek-Górecka, A.; Balwierz, R.; Krawczyk-Łebek, A.; Kostrzewa-Susłow, E.; Bronikowska, J.; Jaworska, D.; Czuba, Z.P. The Effect of Methyl-Derivatives of Flavanone on MCP-1, MIP-1β, RANTES, and Eotaxin Release by Activated RAW264.7 Macrophages. *Molecules* **2024**, *29*, 2239. https://doi.org/10.3390/molecules29102239

Academic Editor: Aziz Hichami

Received: 11 March 2024
Revised: 1 May 2024
Accepted: 8 May 2024
Published: 10 May 2024

Copyright: © 2024 by the authors. Licensee MDPI, Basel, Switzerland. This article is an open access article distributed under the terms and conditions of the Creative Commons Attribution (CC BY) license (https://creativecommons.org/licenses/by/4.0/).

Content

Table S1. Results of pair-wise comparisons of test compounds on the production MCP-1 in compared to control and flavanone in LPS stimulated RAW264.1 cells (n=3). Statistical significance was analysed using Fisher's LSD test. Results marked in red are statistically significant in Fisher's LSD test. Multivariate Tests of Significance ($F = 8.20$, $p < 0.05$).

Table S2. Results of pair-wise comparisons of test compounds on the production MIP-1β in compared to control and flavanone in LPS stimulated RAW264.1 cells (n=3). Statistical significance was analysed using Fisher's LSD test. Results marked in red are statistically significant in Fisher's LSD test. Multivariate Tests of Significance ($F = 8.20$, $p < 0.05$).

Table S3. Results of pair-wise comparisons of test compounds on the production RANTES in compared to control and flavanone in LPS stimulated RAW264.1 cells (n=3). Statistical significance was analysed using Fisher's LSD test. Results marked in red are statistically significant in Fisher's LSD test. Multivariate Tests of Significance ($F = 8.20$, $p < 0.05$).

Table S4. Results of pair-wise comparisons of test compounds on the production eotaxin in compared to control and flavanone in LPS stimulated RAW264.1 cells (n=3). Statistical significance was analysed using Fisher's LSD test. Results marked in red are statistically significant in Fisher's LSD test. Multivariate Tests of Significance ($F = 8.20$, $p < 0.05$)

molecules MDPI

Table S1. Results of pair-wise comparisons of test compounds on the production MCP-1 in compared to control and flavanone in LPS stimulated RAW264.1 cells (n=3). Statistical significance was analysed using Fisher's LSD test. Results marked in red are statistically significant in Fisher's LSD test. Multivariate Tests of Significance ($F = 8.20$, $p < 0.05$).

Cell No.	LSD test; variable MCP-1<br>Probabilities for Post Hoc Tests<br>Error: Between MS = 451,66, df = 26,000<br>sample	{1}	{2}	{3}	{4}	{5}	{6}	{7}	{8}	{9}	{10}	{11}	{12}	{13}
1	DMSO + LPS (5B, 6B)		0.000009	0.000008	0.000043	0.000009	1.000000	0.007050	0.860102	0.081197	0.004759	1.000000	0.201345	0.983816
2	5B 1µM + LPS	0.000009		0.949250	0.569442	0.992730	0.000009	0.016789	0.000015	0.001108	0.024172	0.000009	0.000000	0.000009
3	5B 20µM + LPS	0.000008	0.949250		0.527481	0.956507	0.000008	0.014486	0.000013	0.000939	0.020939	0.000008	0.000000	0.000008
4	6B 1µM + LPS	0.000043	0.569442	0.527481		0.563333	0.000043	0.058434	0.000069	0.004718	0.080639	0.000043	0.000001	0.000041
5	6B 20µM + LPS	0.000009	0.992730	0.956507	0.563333		0.000009	0.016440	0.000015	0.001082	0.023683	0.000009	0.000000	0.000009
6	DMSO + LPS (7B, 8B)	1.000000	0.000009	0.000008	0.000043	0.000009		0.007050	0.860102	0.081197	0.004759	1.000000	0.201345	0.983816
7	7B 1µM + LPS	0.007050	0.016789	0.014486	0.058434	0.016440	0.007050		0.010768	0.276709	0.872748	0.007050	0.000252	0.006711
8	7B 20µM + LPS	0.860102	0.000015	0.000013	0.000069	0.000015	0.860102	0.010768		0.113838	0.007331	0.860102	0.148538	0.844212
9	8B 1µM + LPS	0.081197	0.001108	0.000939	0.004718	0.001082	0.081197	0.276709	0.113838		0.214345	0.081197	0.004334	0.078014
10	8B 20µM + LPS	0.004759	0.024172	0.020939	0.080639	0.023683	0.004759	0.872748	0.007331	0.214345		0.004759	0.000165	0.004526
11	DMSO + LPS (FL)	1.000000	0.000009	0.000008	0.000043	0.000009	1.000000	0.007050	0.860102	0.081197	0.004759		0.201345	0.983816
12	fl 1µM + LPS	0.201345	0.000000	0.000000	0.000001	0.000000	0.201345	0.000252	0.148538	0.004334	0.000165	0.201345		0.208256
13	fl 20µM + LPS	0.983816	0.000009	0.000008	0.000041	0.000009	0.983816	0.006711	0.844212	0.078014	0.004526	0.983816	0.208256	

Table S2. Results of pair-wise comparisons of test compounds on the production MIP-1β in compared to control and flavanone in LPS stimulated RAW264.1 cells (n=3). Statistical significance was analysed using Fisher's LSD test. Results marked in red are statistically significant in Fisher's LSD test. Multivariate Tests of Significance ($F = 8.20$, $p < 0.05$).

LSD test; variable MIP-1ß
Probabilities for Post Hoc Tests
Error: Between MS = 8,6248, df = 26,000

Cell No.	sample	{1}	{2}	{3}	{4}	{5}	{6}	{7}	{8}	{9}	{10}	{11}	{12}	{13}
1	DMSO + LPS (5B, 6B)		0.000497	0.000000	0.024173	0.030536	1.000000	0.014348	0.095306	0.164727	0.008244	1.000000	0.313226	0.243199
2	5B 1µM + LPS	0.000497		0.000086	0.125688	0.103277	0.000497	0.000001	0.000005	0.000012	0.000000	0.000497	0.006679	0.000021
3	5B 20µM + LPS	0.000000	0.000086		0.000001	0.000001	0.000000	0.000000	0.000000	0.000000	0.000000	0.000000	0.000000	0.000000
4	6B 1µM + LPS	0.024173	0.125688	0.000001		0.916189	0.024173	0.000032	0.000337	0.000739	0.000017	0.024173	0.183783	0.001355
5	6B 20µM + LPS	0.030536	0.103277	0.000001	0.916189		0.030536	0.000042	0.000445	0.000973	0.000023	0.030536	0.219120	0.001776
6	DMSO + LPS (7B, 8B)	1.000000	0.000497	0.000000	0.024173	0.030536		0.014348	0.095306	0.164727	0.008244	1.000000	0.313226	0.243199
7	7B 1µM + LPS	0.014348	0.000001	0.000000	0.000032	0.000042	0.014348		0.379970	0.243065	0.815366	0.014348	0.001149	0.164629
8	7B 20µM + LPS	0.095306	0.000005	0.000000	0.000337	0.000445	0.095306	0.379970		0.765542	0.269191	0.095306	0.010466	0.595944
9	8B 1µM + LPS	0.164727	0.000012	0.000000	0.000739	0.000973	0.164727	0.243065	0.765542		0.164513	0.164727	0.020948	0.815677
10	8B 20µM + LPS	0.008244	0.000000	0.000000	0.000017	0.000023	0.008244	0.815366	0.269191	0.164513		0.008244	0.000625	0.107743
11	DMSO + LPS (FL)	1.000000	0.000497	0.000000	0.024173	0.030536	1.000000	0.014348	0.095306	0.164727	0.008244		0.313226	0.243199
12	fl 1µM + LPS	0.313226	0.006679	0.000000	0.183783	0.219120	0.313226	0.001149	0.010466	0.020948	0.000625	0.313226		0.035152
13	fl 20µM + LPS	0.243199	0.000021	0.000000	0.001355	0.001776	0.243199	0.164629	0.595944	0.815677	0.107743	0.243199	0.035152	

Table S3. Results of pair-wise comparisons of test compounds on the production RANTES in compared to control and flavanone in LPS stimulated RAW264.1 cells (n=3). Statistical significance was analysed using Fisher's LSD test. Results marked in red are statistically significant in Fisher's LSD test. Multivariate Tests of Significance ($F = 8.20$, $p < 0.05$).

LSD test; variable RANTES
Probabilities for Post Hoc Tests
Error: Between MS = 231,90, df = 26,000

Cell No.	sample	{1}	{2}	{3}	{4}	{5}	{6}	{7}	{8}	{9}	{10}	{11}	{12}	{13}
1	DMSO + LPS (5B, 6B)		0.329811	0.001264	0.014710	0.213420	1.000000	0.018808	0.171286	0.417540	0.006214	1.000000	0.788692	0.112843
2	5B 1μM + LPS	0.329811		0.014408	0.117247	0.779896	0.329811	0.001701	0.023848	0.080775	0.000505	0.329811	0.217483	0.014022
3	5B 20μM + LPS	0.001264	0.014408		0.325525	0.027235	0.001264	0.000002	0.000032	0.000148	0.000001	0.001264	0.000629	0.000017
4	6B 1μM + LPS	0.014710	0.117247	0.325525		0.192522	0.014710	0.000025	0.000443	0.001989	0.000007	0.014710	0.007782	0.000240
5	6B 20μM + LPS	0.213420	0.779896	0.027235	0.192522		0.213420	0.000825	0.012534	0.045649	0.000241	0.213420	0.134127	0.007202
6	DMSO + LPS (7B, 8B)	1.000000	0.329811	0.001264	0.014710	0.213420		0.018808	0.171286	0.417540	0.006214	1.000000	0.788692	0.112843
7	7B 1μM + LPS	0.018808	0.001701	0.000002	0.000025	0.000825	0.018808		0.281896	0.104542	0.641034	0.018808	0.034223	0.395044
8	7B 20μM + LPS	0.171286	0.023848	0.000032	0.000443	0.012534	0.171286	0.281896		0.564828	0.128360	0.171286	0.266255	0.816784
9	8B 1μM + LPS	0.417540	0.080775	0.000148	0.001989	0.045649	0.417540	0.104542	0.564828		0.040707	0.417540	0.584972	0.421252
10	8B 20μM + LPS	0.006214	0.000505	0.000001	0.000007	0.000241	0.006214	0.641034	0.128360	0.040707		0.006214	0.011841	0.192937
11	DMSO + LPS (FL)	1.000000	0.329811	0.001264	0.014710	0.213420	1.000000	0.018808	0.171286	0.417540	0.006214		0.788692	0.112843
12	fl 1μM + LPS	0.788692	0.217483	0.000629	0.007782	0.134127	0.788692	0.034223	0.266255	0.584972	0.011841	0.788692		0.182341
13	fl 20μM + LPS	0.112843	0.014022	0.000017	0.000240	0.007202	0.112843	0.395044	0.816784	0.421252	0.192937	0.112843	0.182341	

Table S4. Results of pair-wise comparisons of test compounds on the production eotaxin in compared to control and flavanone in LPS stimulated RAW264.1 cells (n=3). Statistical significance was analysed using Fisher's LSD test. Results marked in red are statistically significant in Fisher's LSD test. Multivariate Tests of Significance (F = 8.20, $p < 0.05$).

LSD test; variable eotaxin
Probabilities for Post Hoc Tests
Error: Between MS = 111,89, df = 26,000

Cell No.	sample	{1}	{2}	{3}	{4}	{5}	{6}	{7}	{8}	{9}	{10}	{11}	{12}	{13}
1	DMSO + LPS (5B, 6B)		0.000505	0.001030	0.012593	0.000100	1.000000	0.426578	0.235467	0.508481	0.105781	1.000000	0.934649	0.550337
2	5B 1µM + LPS	0.000505		0.784974	0.208330	0.542086	0.000505	0.003949	0.000021	0.002807	0.000006	0.000505	0.000407	0.000103
3	5B 20µM + LPS	0.001030	0.784974		0.319671	0.379801	0.001030	0.007725	0.000043	0.005545	0.000013	0.001030	0.000832	0.000213
4	6B 1µM + LPS	0.012593	0.208330	0.319671		0.067490	0.012593	0.072398	0.000615	0.054918	0.000184	0.012593	0.010373	0.002912
5	6B 20µM + LPS	0.000100	0.542086	0.379801	0.067490		0.000100	0.000826	0.000004	0.000579	0.000001	0.000100	0.000080	0.000020
6	DMSO + LPS (7B, 8B)	1.000000	0.000505	0.001030	0.012593	0.000100		0.426578	0.235467	0.508481	0.105781	1.000000	0.934649	0.550337
7	7B 1µM + LPS	0.426578	0.003949	0.007725	0.072398	0.000826	0.426578		0.053552	0.891874	0.019784	0.426578	0.381355	0.169558
8	7B 20µM + LPS	0.235467	0.000021	0.000043	0.000615	0.000004	0.235467	0.053552		0.070658	0.648492	0.235467	0.268082	0.547560
9	8B 1µM + LPS	0.508481	0.002807	0.005545	0.054918	0.000579	0.508481	0.891874	0.070658		0.026867	0.508481	0.458069	0.213382
10	8B 20µM + LPS	0.105781	0.000006	0.000013	0.000184	0.000001	0.105781	0.019784	0.648492	0.026867		0.105781	0.123265	0.294196
11	DMSO + LPS (FL)	1.000000	0.000505	0.001030	0.012593	0.000100	1.000000	0.426578	0.235467	0.508481	0.105781		0.934649	0.550337
12	fl 1µM + LPS	0.934649	0.000407	0.000832	0.010373	0.000080	0.934649	0.381355	0.268082	0.458069	0.123265	0.934649		0.605857
13	fl 20µM + LPS	0.550337	0.000103	0.000213	0.002912	0.000020	0.550337	0.169558	0.547560	0.213382	0.294196	0.550337	0.605857	

molecules
